# Insecticide resistance status of malaria vectors in the malaria endemic states of India: implications and way forward for malaria elimination

**DOI:** 10.1016/j.heliyon.2022.e11902

**Published:** 2022-11-26

**Authors:** Kamaraju Raghavendra, Manju Rahi, Vaishali Verma, Poonam Sharma Velamuri, Divya Kamaraju, Kalpana Baruah, Jyoti Chhibber-Goel, Amit Sharma

**Affiliations:** a ICMR-National Institute of Malaria Research (NIMR), Sector 8, Dwarka, Delhi, India; b Indian Council of Medical Research (ICMR), Ramalingaswami Bhavan, New Delhi, India; c National Vector Borne Disease Control Programme, Shastri Park, New Delhi, India; d Molecular Medicine, International Centre for Genetic Engineering and Biotechnology (ICGEB), Aruna Asaf Ali Marg, New Delhi, India

**Keywords:** *Anopheles*, India, Insecticide-resistance, Malaria vectors

## Abstract

**Background:**

In 2012, the World Health Organization (WHO) released the Global Plan for Insecticide Resistance Management in malaria vectors to stress the need to address insecticide resistance. In a prospective multi-centric study commissioned by the Indian Council of Medical Research (ICMR), we assessed the insecticide susceptibility status of the primary malaria vectors in India from 2017 through 2019.

**Methods:**

The insecticide susceptibility status of the prevalent primary malaria vectors – *An. culicifacies, An. fluviatilis, An. stephensi, An. minimus* and *An. baimaii* and secondary malaria vectors - *An. aconitus, An. annularis* and *An. philippinensis/nivepes* from 328 villages in 79 districts of 15 states of India were assessed following the WHO method mainly to insecticides used in vector control, organochlorine (DDT), organophosphate (malathion), and other pyrethroids (alpha-cypermethrin, cyfluthrin, lambda-cyhalothrin and permethrin). The study sites were selected as suggested by the National Vector Borne Disease Control Programme.

**Results:**

The primary malaria vector *An. culicifacies* showed resistance to DDT (50/50 districts including two districts of Northeastern India), malathion (27/44 districts), and deltamethrin (17/44 districts). This species was resistant to DDT alone in 19 districts, double resistant to DDT-malathion in 16 districts, double resistant to DDT-deltamethrin in 6 districts, and triple resistant to DDT-malathion-deltamethrin in 9 districts. *An. minimus* and *An. baimaii* were susceptible in Northeastern India while *An. fluviatilis* and the secondary malaria vector *An. annularis* was resistant to DDT in Jharkhand.

**Conclusion:**

In this study we report that among the primary vectors *An. culicifacies* is predominantly resistant to multiple insecticides. Our data suggest that periodic monitoring of insecticide susceptibility is vital. The national malaria program can take proactive steps for insecticide resistance management to continue its push toward malaria elimination in India.

## 1 Introduction

The World Malaria Report 2021 estimated ∼241 million malaria cases worldwide in 2020, with India accounting for ∼83% of the total malaria cases -i.e ∼4.8 million within the WHO South-East Asia region, and a maximum region-wide decrease in the number of cases was registered, with 23 million in 2000 to about 5 million in 2020 [1]. Insecticides have been the mainstay of vector control in India and elsewhere among various vector control strategies [2, 3]. Currently, 83 vector control products have been prequalified by World Health Organization vector control product prequalification system [4]. In India, currently, the following insecticides are deployed against malaria vectors – (1) for control of adult malaria vector - dichloro-diphenyl-trichloroethane (DDT), malathion, and five pyrethroid insecticides (alpha-cypermethrin, bifenthrin, cyfluthrin, deltamethrin, lambda-cyhalothrin); (2) for larval control – temephos, two bacterial pesticides (*Bacillus thuringiensis* var. israelensis*, B. sphaericus*), two insect growth regulator (IGR) compounds (diflubenzuron, pyriproxyfen), and (3) for space spray, malathion (technical) and formulations of deltamethrin, cyphenothrin (synthetic pyrethroid) or natural pyrethrum extract [5]. For control of adult malaria vectors deltamethrin and alpha-cypermethrin impregnated long-lasting insecticidal nets (LLINs) are used [2]. A comprehensive analysis of insecticide resistance data from India between 1991 to 2016 indicated an increase in resistance and the potential to derail the progress made in reducing malaria transmission [6]. The present study was conducted from 2017 to 2019 in the select districts suggested by the National Vector Borne Disease Control Programme (NVBDCP) that had lacked insecticide resistance data in recent years. The data have been generated for some districts after a gap of a decade or more.

WHO encourages countries to develop local data-based insecticide resistance management plans. Data on insecticide resistance, including the frequency and intensity of malaria vectors and the resistance mechanism, is scarce from malaria-endemic regions. According to the World Malaria Report 2021, of the 88 countries that have provided data on insecticide resistance from 2010 to 2020, ∼80% of countries reported resistance to pyrethroids, ∼64% to organochlorines, ∼34% to carbamates, and ∼28% countries to organophosphates [1]. Further, reports on Global Insecticide Resistance (2010–16) have indicated an increased intensity and time to kill 50% of the exposed mosquitoes [7].

India has six primary malaria vectors – *An. culicifacies*, *An. fluviatilis*, *An. stephensi*, *An. baimaii*, *An. minimus* and *An. sundaicus*; and four secondary malaria vectors – *An. annularis*, *An*. *philippinensis/nivepes*, *An*. *jeyporiensis* and *An*. *varuna.* [8] Amongst these, *An. culicifacies* (rural and peri-urban areas) contributes ∼67%, *An. fluviatilis* (forested areas) ∼15% and *An. stephensi* (urban areas) ∼12% of the malaria cases in India [6]. Annually, ∼93% of the malaria cases were contributed by these three primary malaria vector species and, ∼7% of cases are from *An. minimus* (foothills in the east), *An. baimaii* (forested areas in northeastern states) and *An. sundaicus* (coastal areas of the Andaman and Nicobar Islands).

Malaria is endemic in 36 states/UTs in India. The Indian Council of Medical Research (ICMR) commissioned multi-centric field studies from 2017 to 2019 to determine the insecticide resistance status of primary and secondary malaria vectors across India. The study was conducted in 15 states as suggested by the NVBDCP. The states were Odisha, Jharkhand, Madhya Pradesh, Karnataka, Maharashtra, Gujarat, Haryana, Uttar Pradesh, and 7 Northeastern Indian states – Arunachal Pradesh, Assam, Manipur, Meghalaya, Mizoram, Nagaland, and Tripura. The districts suggested in these states reportedly lacked recent insecticide susceptibility data for major malaria vectors. Our data sheds light on the insecticide susceptibility in vectors during the years 2017–2019 across India and has implications for the ongoing efforts at malaria elimination in India.

## 2 Materials and methods

### 2.1 Study area

**Figure 1:**
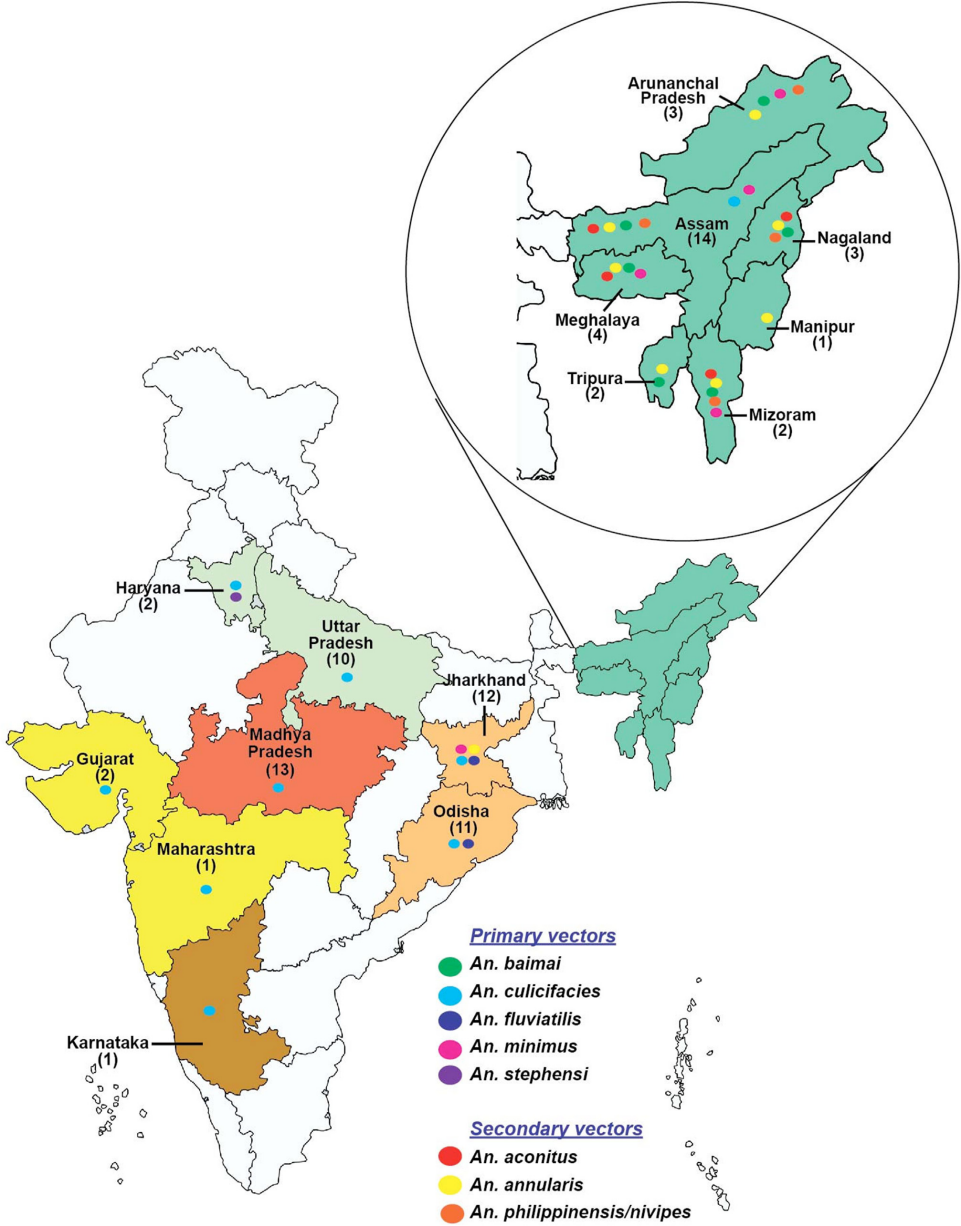
The distribution of the primary and secondary *Anopheles* vectors in several districts (shown in brackets) from the fifteen states for insecticide resistance studies.

The multi-centric field-based insecticide susceptibility study covered 328 villages in 79 districts under 15 Indian states to ensure inclusion of major topographies (forest, forested hills, plains, and coastal areas) and different malaria eco-epidemiological settings (Table 1). Susceptibility to different classes of insecticides was determined for prevalent primary malaria vectors – *An. culicifacies, An. minimus, An. baimaii* in the Northeastern states in India and *An. culicifacies*, *An. fluviatilis* and *An. stephensi* in other Indian states and additionally to secondary malaria vectors, *An. aconitus, An. annularis and An. philippinensis/nivepes* (Figure 1). Mosquitoes were mainly exposed to insecticides in use in vector control in Indian vector control programme, DDT, malathion and deltamethrin/alpha-cypermethrin/lambda-cyhalothrin/cyfluthrin and in some locations to other WHO-approved insecticides for use in vector control namely, fenitrothion and permethrin. The data on vector control interventions, i.e., DDT/SP-IRS and LLINs employed in the 13 Indian states, except that Odisha (coastal) and Uttar Pradesh which received focal spray with DDT-IRS, were compiled (Table 1).

**Table 1:** Table showing states, districts surveyed, geolocations, interventions, number of villages surveyed, topography, primary and secondary vectors.

S. No.	State (location in India)	District	Location in the state	Geolocation	Vector intervention/s (adult) last 5 years	No. of Villages surveyed	Villages (Topography)	Primary and Secondary vector Species
**1**	**Odisha (East India)**	Kalahandi	South	20.083°N 83.2°E	DDT-IRS & LLIN	4	Plain, Foothill, Forest	Primary -*An. culicifacies, An. fluviatilis*
		Angul	Central	20°50′17″N ​85°05′44″E		4		
		Cuttack	East	20°31′25″N ​85°47′17″E	Focal Spray	4	Coastal	
		Khurda	East	20.18°N 85.62°E		4		
		Puri	East	19°48′38″N ​85°49′53″E		4		
		Bhadrak	East	21.06°N 86.50°E		4		
		Jagatsinghpur	East	20.266°N 86.166°E		4		
		Balasore	East	21.5°N 86.9°E		4		
		Kendrapara	East	20.50°N 86.42°E		4		
		Jajpur	East	20.85°N 86.33°E		4		
		Mayurbhanj	East	21.933°N 86.733°E		4		
**2**	**Jharkhand (East India)**	Simdega	South	22.62°N 84.52°E	DDT-IRS & LLIN	6	Plain, Foothill, Hills, Hilltop	Primary*- An. culicifacies, An. fluviatilis,* Secondary-*An. annularis* (local importance)*, An. minimus*
		Gumla	South	23°N 84.50°E		8	Plain, Forest, Hilltop	
		Khunti	South	23.0140203°N 85.2724457°E		5	Plain, Forest, Foothill, Hills	
		West Singhbum	South	21.97°N 86.90°E		7	Forest, Hills	
		Giridih	North	24°10′48″N 86°19′12″E		8	Plain, Foothill, Hills	
		Latehar	West	23°43′48″N ​84°28′12″E		7	Foothill, Forest	
		Palamu	North	24°1′48″N ​84°4′12″E		8	Plain, Forest	
		Koderma	North	24.47°N 85.6°E		9		
		Chatra	North	24°12 ​0″N ​84°52′12″E		6	Plain, Foothill	
		Dhanbad	East	23°47′24″N ​86°25′48″E		6		
		Godda	East	24.83°N 87.22°E	SP-IRS & LLIN	4	Plain, Forest, Foothill	
		Sahibganj	East	25.25°N 87.65°E		5	Plain, Foothill, Hills	
**3**	**Madhya Pradesh (Central India)**	Umaria	East	23°31′36.65″N ​80°50′17.18″E	DDT-IRS	6	Plain, Foothill, Hilltop	Primary *-An. culicifacies*
		Bhind	North	26°33′51.12″ ​N ​78°47′17.88″E		7	Plain, Foothill	
		Anuppur	East	23.1°N 81.68°E	DDT-IRS & LLIN	6	Plain, Foothill, Hilltop	
		Khargone	South	21°49′23″N ​75°36′37″E	SP-IRS	6		
		Shivpuri	North	25.43°N 77.65°E		7		
		Hoshangabad	South	22°40′0″N ​77°30′0″E	SP-IRS & LLIN	6		
		Panna	East	24°43′12″N ​80°10′48″ ​E		6		
		Singrouli	East	24°12′0″N ​82°40′12″E		6		
		Alirajpur	West	22°18′18″N ​74°21′36″E		6		
		Dhar	West	22°36′0″N ​75°18′0″E		6		
		Sidhi	East	24°25′0.12″N 81°52′59.88″E		8	Plain, Forest, Foothill	
		Tikamgarh	North	24°44′24″N ​78°49′48″E	(NI)	3	Plain, Forest	
		Datia	North	25°40′12″N ​78°27′36″E		6	Plain, Forest	
**4**	**Karnataka (South India)**	Kalaburgi	North	17.33°N 76.83°E	Malathion & SP - IRS & LLIN	3	stone quarry, irrigation area, riverine	Primary -*An. culicifacies, An. fluviatilis*
**5**	**Maharashtra (West India)**	Gadchiroli	East	20°11′10″N ​80°00′19″E	SP-IRS & LLIN	12	Forest, Foothill, Plain	Primary -*An. culicifacies*
**6**	**Gujarat (West India)**	Kheda	Central	22°45′0″N ​72°41′0″E	SP-IRS & LLIN	4	Riverine and canal irrigated plain	Primary -*An. culicifacies*
		Panchmahal	Central	22°45′N ​73°36′E		4		
**7**	**Uttar Pradesh (North India)**	Gautam Budh Nagar	North	28.34°N 77.19°E	DDT-IRS	7	Plain	Primary -*An. culicifacies*
		Saharanpur	North	29.97°N 77.55°E	DDT-IRS-Focal	9		
		Badaun	Central	28.2°N 79.7°E		9		
		Hathras	West	27.26°N 78.3°E		10		
		Jhansi	South	25.25°N 78.34°E		13		
		Banda	South	25.30°N 80.30°E		14		
		Kanpur Dehat	Central	26.24°N 79.59°E		16		
		Prayag Raj	South	25.27°N 81.50°E		13	Rocky	
		Mirzapur	South	27.40°N 29.33°E		15	Forest, Hills	
		Sonebhadra	South	24.42°N 83.4°E		12		
**8**	**Haryana (North India)**	Nuh	South	28°6′0″N ​77°0′0″E	DDT-IRS	3	Plain and agriculture land	Primary -*An. culicifacies, An. stephensi*
		Palwal	South	28.143°N 77.329°E		3		
**9**	**Arunachal Pradesh (North East India)**	Changlang/3	South	27°7′48″N ​95°44′24″E	DDT-IRS & LLIN	3	Hilltop, Plains	Primary - *An. annularis,* Secondary -*An. minimus, An. philippinensis/nivipes, An. baimaii*
		Tirap	South	26°59′26.52″N ​95°30′10.08″E		3	Hilltop, Foothill	
		Namsai	South	27.6689°N 95.8714°E		5	Hill, Hilltop, Forested Hill	
**10**	**Assam (North East India)**	Kokrazhar	West	26° ​24′ ​0″ ​N, ​90° ​16′ ​12″ ​E	DDT-IRS & LLIN	1	Plain Forest	Primary - *An. culicifacies, An. annularis,* Secondary - *An. minimus,An. philippinensis/nivipes, An. baimaii,An. aconitus*
		Baksa	North	26° ​34′ ​51″ ​N, ​91° ​25′ ​13″ ​E		1		
		Bongaigaon	West	26.4667°N 90.5667°E		1		
		Kamrup (M)	West	26°11′0″N 91°44′0″E		2	Foothills	
		Goalpara	West	26° ​26′ ​0″ ​N, ​90° ​22′ ​0″ ​E		1		
		Udalguri	North	26° ​44′ ​42.72″ ​N, ​92° ​5′ ​46.32″ ​E		5	Plain forest, foothills	
		Kamrup (R)	West	26°20′N ​91°15′E		1	Plain	
		Nalbari	West	26° ​27′ ​0″ ​N, ​91° ​26′ ​24″ ​E		1		
		Tinsukia	East	27.500°N 95.367°E		2		
		Dibrugarh	East	27.48&deg;N 95&deg;E		4	Forested foothill, Forested Plains, Foothill	
		Jorhat	East	26.75&deg;N 94.22&deg;E		4	Forested foothill, Foothill, Plain, Forested Plain	
		Golaghat	East	26° ​0′ ​0″ ​N, ​93° ​0′ ​0″ ​E		6	Forested Plain, Foothill, Plain	
		KarbiAnglong	Central	26° ​11′ ​0″ ​N, ​93° ​34′ ​0″ ​E		3	Forested foothill, Forested Plain, Hilltop	
		Sivasagar	East	26.98°N 94.63°E		6	Plain, Foothill	
**11**	**Manipur (North East India)**	Temenglong	West	24°59′26.39″N ​93°30′3.26″E	DDT-IRS & LLIN	6	Hilltop, Foothill, Plain	Secondary -*An. annularis*
**12**	**Meghalaya (North East India)**	Ri Bhoi	North	25° ​54′ ​0″ ​N, ​91° ​53′ ​0″ ​E	DDT-IRS & LLIN	1	Foothills	Primary - *An. baimaii,* Secondary -*An. annularis,An. minimus,An. philippinensis/nivipes*
		South West Garo Hills	South			1		
		EastGaro Hills	West	25° ​29′ ​43.66″ ​N, ​90° ​37′ ​0.55″ ​E		1		
		South Garo Hills	South	25° ​12′ ​0″ ​N, ​90° ​38′ ​0″ ​E		7	Foothill, Hilltop	
		West Garo Hills	West	25° ​31′ ​0.12″ ​N, ​90° ​13′ ​0.12″ ​E		6		
**13**	**Mizoram**	Kolasib	North	24°13′48″N ​92°40′48″E	DDT-IRS & LLIN	8	Foothill, Forested Plain, Hilltop, Forested foothill, Plain, Forested Hilltop	Primary -*An. minimus*, Secondary - *An. annularis, An. philippinensis/nivipes, An. aconitus*
		Mamit	West	23.9294° N, 92.4906° E		4	Hilltop, Plain	
**14**	**Nagaland (North East India)**	Mokokchung	North	26° ​19′ ​12″ ​N, ​94° ​30′ ​0″ ​E	DDT-IRS & LLIN	15	Hilltop, Foothill, Forested Plain, Plain, Forested Hilltop	Primary *-An. baimaii,* Secondary - *An. annularis, An. philippinensis/nivipes, An. aconitus*
		Dimapur	West	25.92&deg;N 93.73&deg;E		6	Foothill, Plain, Hilltop	
		Peren	South	25&deg;31&prime;N&nbsp;93&deg;44&prime;E		4	Plain, Foothill	
**15**	**Tripura (North East India)**	Dhalai	East	23° ​56′ ​0″ ​N, ​91° ​51′ ​0″ ​E	DDT-IRS & LLIN	5	Forested hill, Foothill	Primary - *An. baimaii* Secondary -*An. annularis,*
		South Tripura	South	23° ​32′ ​0″ ​N, ​91° ​29′ ​0″ ​E		5	Plain

### 2.2 Mosquito collection

Mosquitoes were collected following standard WHO methods [9]. In the districts selected for surveillance, villages with good mosquito productivity were identified. A team of ∼3 people collected mosquitoes from human dwellings and cattle sheds. *Anophelines* resting indoors in human habitats and cattle holding sheds were collected by hand catch using an aspirator and torch in the early morning hours (0600 h–0800 h), spending 15 min per structure. The collected mosquitoes were transferred to a cloth cage and brought to the laboratory (wrapped in a wet towel). The mosquitoes were identified based on specific morphological characteristics, and female vector species were separated and used for susceptibility tests.

### 2.3 Insecticide susceptibility tests

Mixed-age field-collected preferably full-fed stage mosquitoes were used in the study. Primarily, mosquitoes were exposed to insecticides in use in vector control in public health; namely DDT, malathion, and deltamethrin, and exposure to other insecticides was made to assess susceptibility to insecticides in the same or different classes of insecticides approved for vector control for more qualitative data. These tests were conducted using the standard WHO methods and kits to WHO specified discriminatory dosages of insecticide-impregnated papers. These kits and insecticide-impregnated and control papers were procured from Universiti Sains Malaysia (USM), Penang, Malaysia. Four replicates of 20–25 female mosquitoes were exposed for 1 h (except fenitrothion for 2 h) to the WHO prescribed discriminating dosages of insecticides (DDT 4%, malathion 5%, fenitrothion 1%, deltamethrin 0.05%, alpha-cypermethrin 0.05%, permethrin 0.75%, cyfluthrin 0.15%, lambda-cyhalothrin 0.05%). Two control replicates of 20–25 female mosquitoes were run simultaneously alongside the test replicates (Supplementary Table 1). However, fewer mosquitoes were exposed in case of species having low densities. The susceptibility tests were conducted in a base laboratory in the field at the prescribed temperature (27 ± 2 °C) and humidity conditions (75 ± 10%). After 24 h, the dead and alive mosquitoes were scored, and percent mortality in test and control replicates was determined.

The states, districts and villages included in the study and their respective geolocations and vector control intervention strategies are listed in Table 1. The number of adult mosquito vector species collected to determine the percentage mortality and susceptibility to different insecticides for each state is briefly mentioned below and provided in Supplementary Table 1A–D.

In the state of Odisha, *An. culicifacies* (n = 1380) and *An. fluviatilis* (n = 800) were exposed to DDT, malathion, deltamethrin and cyfluthrin in 9 districts (three ecotypes namely plain, foothill and forest). In the state of Jharkhand, the insecticide susceptibility status to different classes of insecticides was determined for three primary malaria vectors – *An. culicifacies* (n = 10,194; 12 districts), *An. fluviatilis* (n = 3155; 9 districts), *An. minimus* (n = 120; 1 district) and secondary malaria vector *An. annularis* (n = 2500; 6 districts). Collections were made from five major ecotypes (plain, forest, foothill, hilly, and hilltop). The insecticide susceptibility data in Madhya Pradesh were determined only for major malaria vector *An. culicifacies* (n = 5740) from 13 districts with four major ecotypes (plain, forest, foothill and hilltop). *An. culicifacies* (n = 203) was studied for insecticide resistance in the district Kalaburgi of Karnataka. Insecticide susceptibility studies for *An. culicifacies* (n = 1490) were performed in the district Gadchiroli of Maharashtra with three major ecotypes (plain, foothill, and forest topographies). Studies were conducted for *An. culicifacies* (n = 1280) in two districts, Kheda and Panchmahal of Gujarat state for malathion, deltamethrin, permethrin, and alpha-cypermethrin. *An. culicifacies* (n = 805) and *An. stephensi* (n = 795) were tested for insecticide susceptibility status in 2 districts (Nuh and Palwal) with plain and agricultural ecotypes from Haryana. Susceptibility status of *An. culicifacies* (n = 5030) was determined in 10 districts of Uttar Pradesh state with three different ecotypes (plain, rocky, hilly forested). Prayagraj with a rocky terrain; Mirzapur and Sonebhadra both with hilly-forested terrain, and the rest of seven districts had plain terrain. Studies were conducted in 29 districts of 7 Northeastern states against two primary malaria vectors *An. minimus* and *An. baimaii* and in Assam at two locations against *An. culicifacies*. The major topography in different states was mostly forested and in some plain ecotype. Insecticide susceptibility tests were conducted against DDT, fenitrothion, malathion, deltamethrin, lambda-cyhalothrin, and permethrin. Besides these, malaria vectors of secondary importance - *An. annularis*, *An. philippinensis/nivipes* and *An. aconitus* were tested in some states, mostly Northeastern states, depending upon their prevalence.

### 2.4 Data analysis

The pooled data of the test replicates for a given insecticide were compiled and analyzed.

Percent mortality in the insecticide susceptibility tests was calculated using the formula:

$$\%\;\text{mortality}\;\left(\text{Test}\;\text{and}\;\text{Control}\right)=\frac{\text{Number}\;\text{of}\;\text{dead}\;\text{mosquitoes}}{\text{Number}\;\text{of}\;\text{mosquitoes}\;\text{exposed}\;}\times 100\%$$

Tests with control mortality exceeding 20% were discarded. If the mortality in control was found 5%–20%, then the mortality in the test was corrected using Abbott's formula [10],

$$\%\;Corrected\;\text{mortality}=\frac{\%\;Test\;\text{mortality}-\%\;Control\;\text{mortality}}{100-\%\;Control\;\text{mortality}\;}\times 100\%$$

The susceptibility status of the mosquito species against the tested insecticide is categorized as: (1) susceptible (98%–100% mortality); (2) possible resistance (90%–97% mortality), and (3) confirmed resistance (<90% mortality) [9].

### 2.5 Statistical analysis

Mosquito mortality data were subjected to Chi-square/Fisher exact tests using the SPSS Software package (Ver 20) to assess possible correlation.

### 2.6 Patient and public involvement

Patients were not involved in the current study.

## 3 Results

A total of 43,862 female malaria vector mosquitoes (primary and secondary) belonging to *An. culicifacies*, *An. fluviatilis*, *An. stephensi*, *An. baimaii*, *An. minimus*, *An. aconitus, An. annularis*, *An*. *philippinensis/nivepes* were exposed to insecticides of different classes. State-wise details and results are given below.

### 3.1 State of Odisha

**Figure 2:**
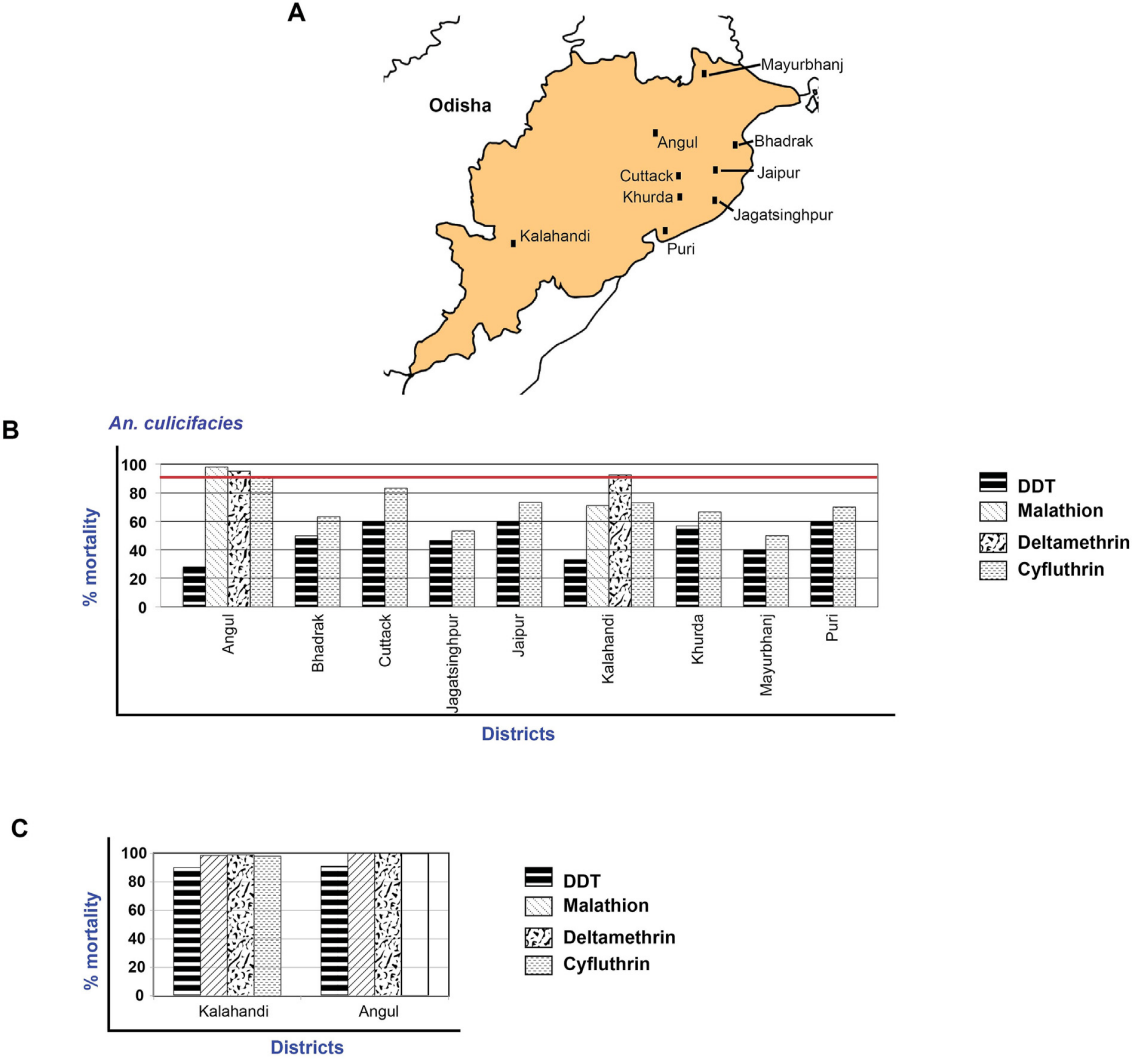
Map of Odisha showing the location of study districts (A) and susceptibility status of *An. culicifacies* (B) and *An. fluviatilis* (C). % mortality of 98-100 % suggests the vector species are susceptible to the insecticide (shown in red line).

*An. culicifacies* was resistant to DDT (% mortality – 30%–60%) and cyfluthrin (% mortality – 50%–90%) in all the nine districts (Figure 2A). *An. culicifacies* was possibly resistant to deltamethrin in both Angul and Kalahandi. On the other hand, it was susceptible to malathion in Angul and resistant to malathion in Kalahandi (mortality – 70%) (Figure 2B). *An. fluviatilis* was susceptible to malathion, cyfluthrin, and deltamethrin in the two tribal districts (mortality – 98–100%) but possible resistant to DDT (mortality – 90–91%) (Figure 2C).

### 3.2 State of Jharkhand

**Figure 3:**
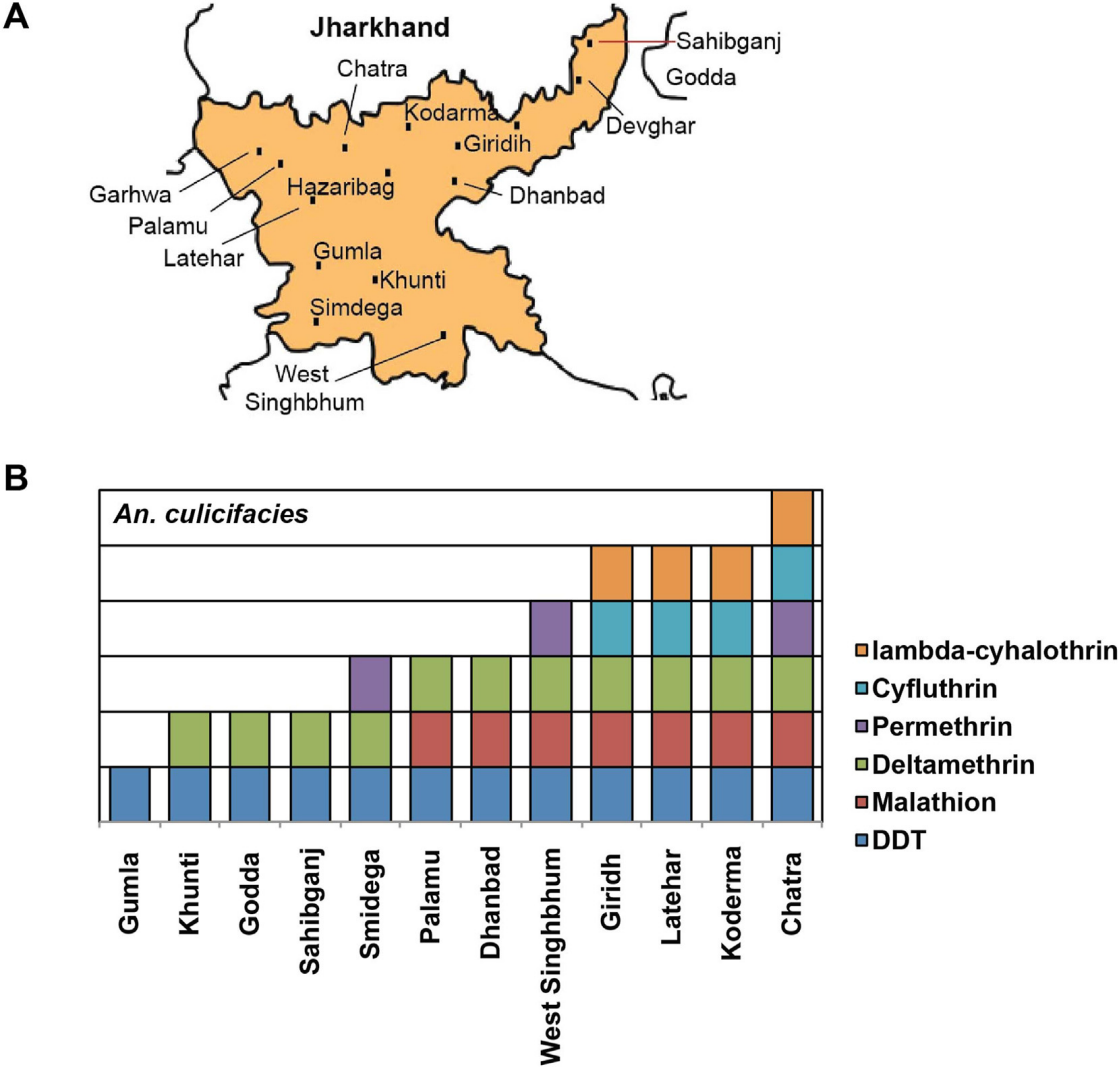
Map of Jharkhand showing the location of study districts (A) and susceptibility status of *An. culicifacies* to different insecticides in different districts (B).

*An. culicifacies* was found resistant to DDT in the surveyed 12 districts (Figure 3A) (mortality – 4.5%–47%), to malathion in 6 (mortality – 69%–89.5%); and to deltamethrin in 10 districts (mortality – 58.5%–87.5%) (Supplementary Table 1A, Figure 3B). Further, *An. culicifacies* was resistant to permethrin in 5 districts (mortality – 65.5%–89%), lambda-cyhalothrin in 4 districts, and cyfluthrin in 3 districts (mortality 40%–86.5%) (Supplementary Table 1A, Figure 3B). Possible resistance was recorded in one district each for malathion, deltamethrin and lambda-cyhalothrin, in two districts for cyfluthrin, and five districts for permethrin (Supplementary Table 1A). The observed insecticide susceptibility data from 12 districts for six insecticides were analyzed for correlation with five ecotypes. A significant association was found in all the five ecotypes, and the data for each district with correlation status is listed in Supplementary Table 2. More importantly, category-wise analysis of insecticide resistance data of *An. culicifacies* (confirmed resistance, possible resistance, and susceptible) to find a correlation with topographies was found significant for malathion only (p = .037, p < .05).

*An. fluviatilis* was found resistant to DDT in all the nine districts (mortality range - 18.5%–80%) and susceptible to malathion, deltamethrin, and permethrin in 6 districts (Supplementary Table 1B). The study districts represented a combination of 2 major topographies of the 5, namely: plain, forest, foothill, hilly, and hilltop. Studies were undertaken in Simdega and Gumla that represented plain and hilltop, Khunti, Palamu (plain and forested), Sahibganj, Giridih (plain and foothills), West Singhbhum (forested and hilly) and Godda and Latehar (forested and foothills; Table 1). *An fluviatilis* susceptibility data for all the insecticides was statistically not significant for correlation between mortality rate and ecotypes, unlike the case for *An. culicifacies.* The insecticide susceptibility studies were conducted in 6 districts for the secondary malaria vector *An. annularis. An. annularis* was found susceptible to DDT, malathion, deltamethrin, and permethrin in the surveyed districts. At the same time, *An. annularis* showed possible resistance in 3 districts of Jharkhand towards malathion and confirmed resistance in 6 districts of Jharkhand towards DDT (Supplementary Table 1D). The other important vector *An. minimus* was possible resistant to DDT and susceptible to malathion, deltamethrin, and permethrin (Supplementary Table 1C).

### 3.3 State of Madhya Pradesh

**Figure 4:**
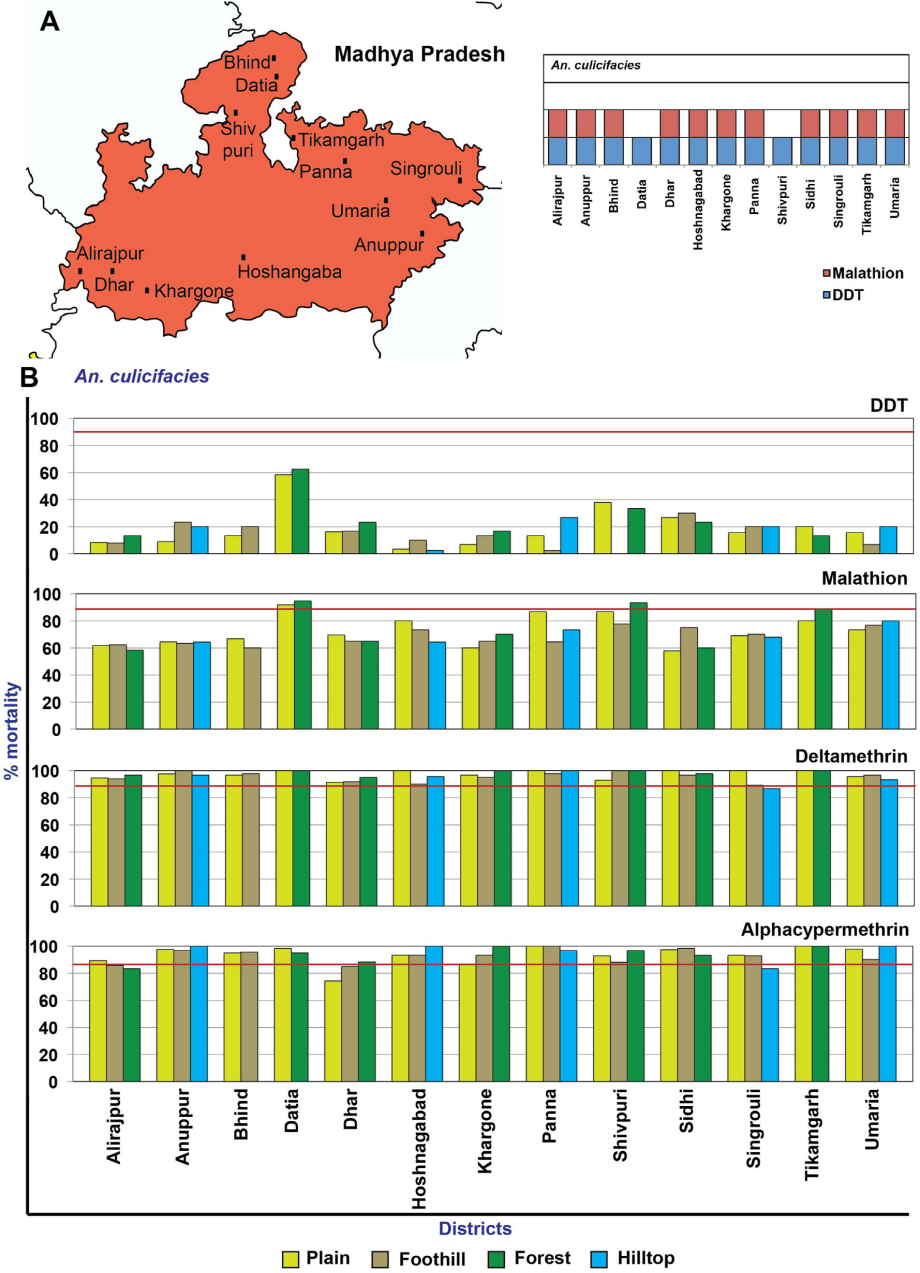
Map of Madhya Pradesh showing the location of study districts and the *An. culicifacies* resistant insecticides represented by a box for each district (A), % mortality of the *An. culicifacies* to different insecticides in different districts (B).

The pooled data on susceptibility to the three insecticides (DDT, malathion, and alpha cypermethrin) from different villages in the four topographies did not show an association (p < 0.05). *An. culicifacies* was observed as resistant to DDT (in 13 districts) (Figure 4A) (mortality – 7.6%–60%), to malathion (in 12 districts) (mortality – 58.1%–87%) (except Datia, where the species showed possible insecticide resistance), while to alpha-cypermethrin (in 2 districts) (Dhar and Alirajpur) (mortality – 82.9% and 85.7%, respectively). The species was susceptible to alpha-cypermethrin in 3 districts while it showed possible resistance in 8 districts. *An. culicifacies* was possible resistant to deltamethrin in 8 districts but was susceptible in 5 districts (Figure 4B).

### 3.4 State of Karnataka

**Figure 5:**
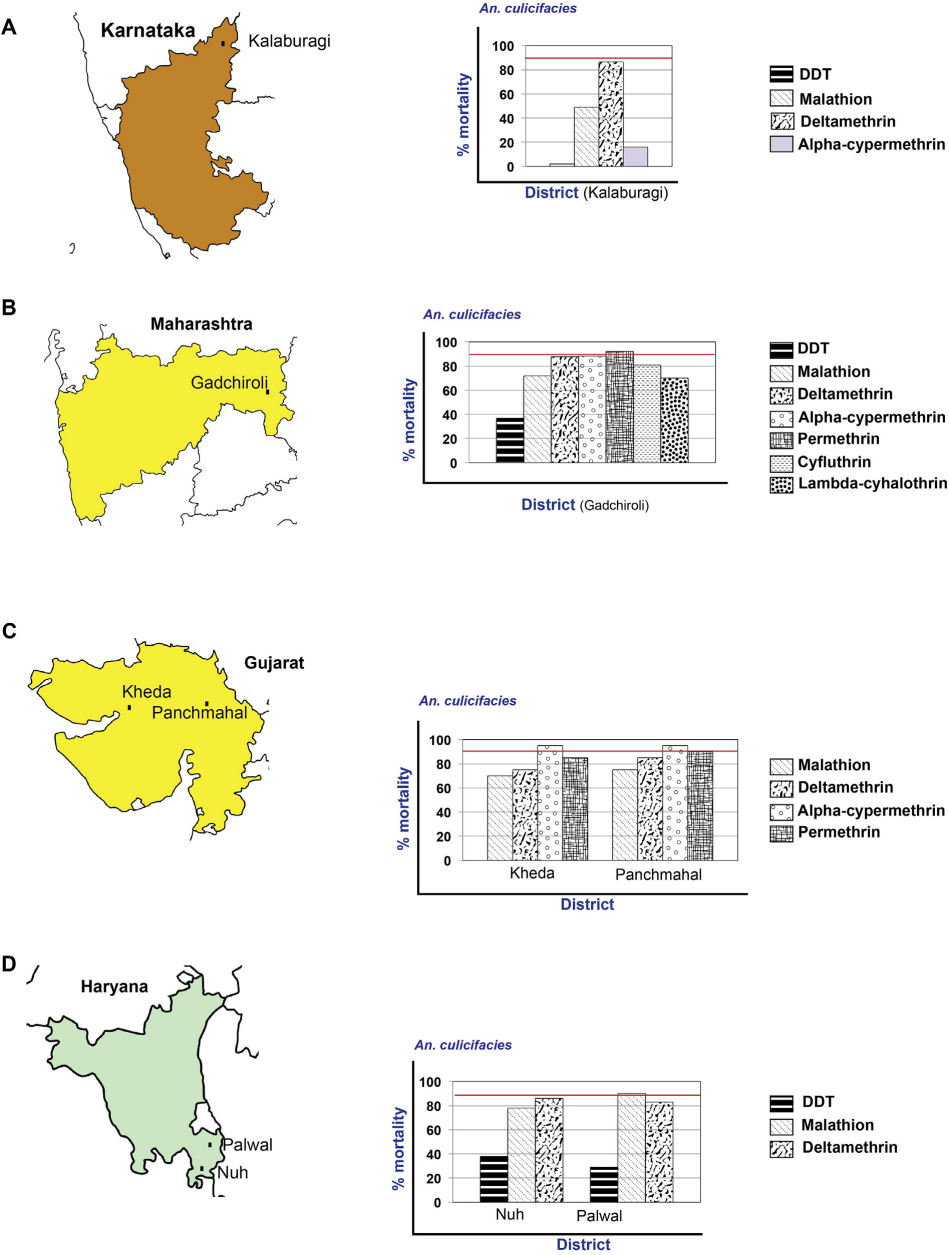
Map of Karnataka (A), Maharashtra (B), Gujarat (C), and Haryana (D), showing the location of study districts and susceptibility status of *An. culicifacies* to different insecticides in different districts in the states. % mortality of 90% suggests the vector species are susceptible to the insecticide (shown in red line).

*An. culicifacies* was found resistant to all the four insecticides studied - DDT (mortality – 2%), malathion (mortality – 49%), deltamethrin (mortality – 86.5%) and alpha-cypermethrin (mortality – 16%) (Figure 5A). The data were generated from single terrain of stone quarry area.

### 3.5 State of Maharashtra

Data showed that *An. culicifacies* was resistant to the six insecticides used in India, i.e., DDT (mortailty – 37%), malathion (mortailty – 71.9%), deltamethrin (mortailty – 87.8%), alpha-cypermethrin (mortailty – 88.2%), cyfluthrin (mortailty – 81%), and lambda-cyhalothrin (mortailty – 70.1%), with possible resistance to permethrin (mortality – 92.2%) (Figure 5B).

### 3.6 State of Gujarat

*An. culicifacies* was resistant to malathion in Kheda and Panchmahal (mortality – 70% and 75%) and deltamethrin (mortality – 75% and 85%). In Kheda, *An. culicifacies* was resistant to permethrin, while in Panchmahal, it showed possible resistance (mortality – 85% and 90%). It showed possible resistance to alpha-cypermethrin in both the districts (mortality – 95%) (Figure 5C). Both the districts represented the common topography of the riverine and canal irrigated plain. The susceptibility status to the four insecticides (malathion, deltamethrin permethrin, and alpha-cypermethrin) was not significant at p < 0.05.

### 3.7 State of Haryana

In both the districts (Nuh and Palwal) *An. culicifacies* was resistant to DDT (mortality – 38% and 29%), malathion (mortality – 78% and 90%) and deltamethrin (mortality – 86% and 83%) (Figure 5D). Similarly, *An.stephensi* was resistant to DDT (mortality – 31% and 48%, respectively), malathion (mortality – 69 and 81%) and to deltamethrin (mortality – 85% and 72%, respectively) (Figure 5D). Both the districts have common topography, and the mortalities in *An. culicifacies* with DDT and deltamethrin were not significant, while they were significant for malathion at p < 0.05. For *An. stephensi,* the mortalities were significant for all three insecticides at p < .05 (see Supplementary Table 2).

### 3.8 State of Uttar Pradesh

**Figure 6:**
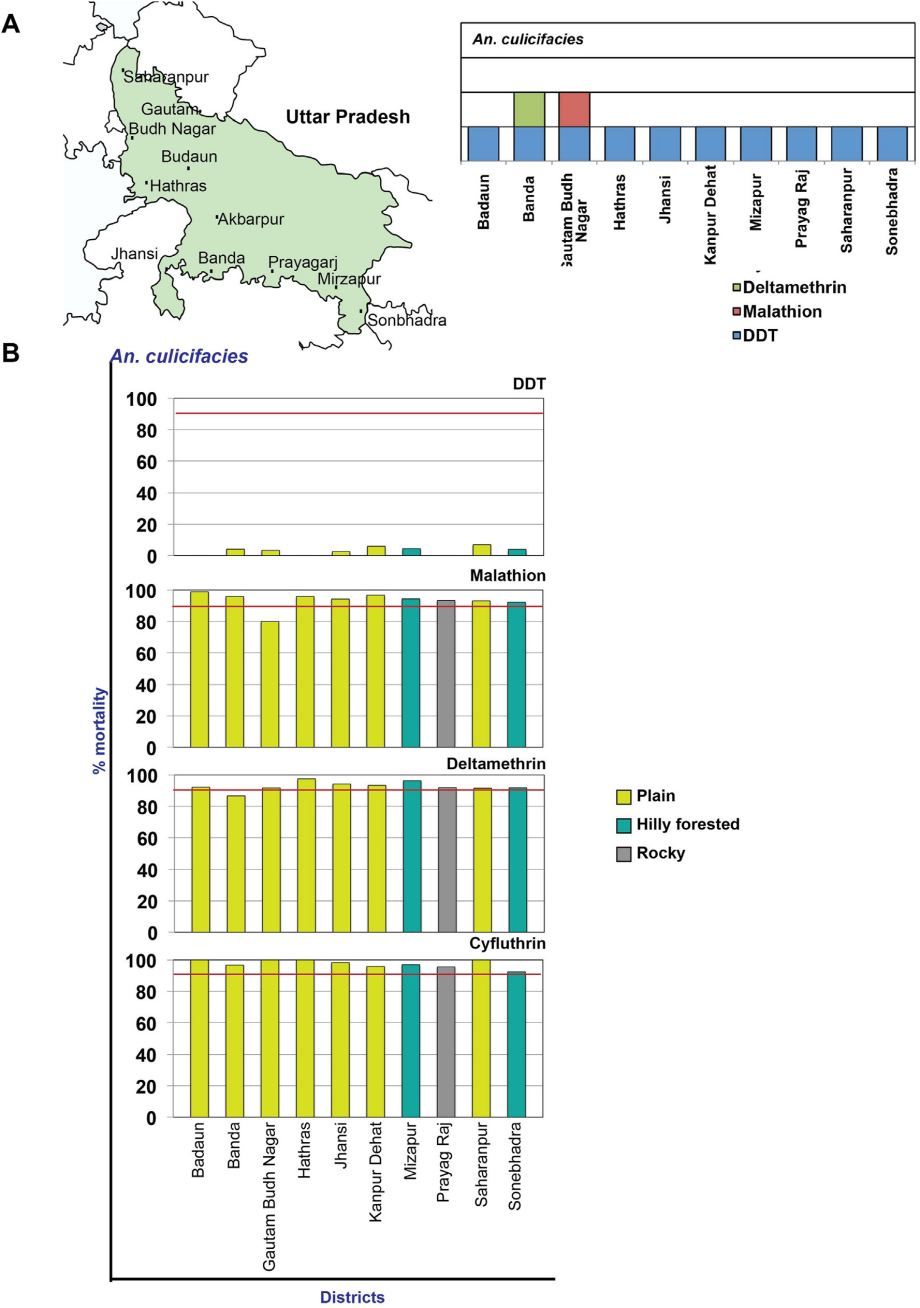
Map of Uttar Pradesh showing the location of study districts and the *An. culicifacies* resistant insecticides represented by a box for each district (A), % mortality of the *An. culicifacies* to different insecticides in different districts (B).

*An culicifacies* in Uttar Pradesh was found resistant to DDT in all the ten districts (% mortality – 0 to 6.9% (Figure 6A and B). In 8 districts, this species was in the possible resistance category to malathion (mortality – 92.2%–96.7%), but was resistant to malathion in Gautam Budh Nagar district and susceptible in the district Badaun with 98.9% mortality. For deltamethrin, the species showed possible resistance in 10 districts with a mortality range of 91.5%–97.5%, while *An culicifacies* was resistant in the Banda district, registering 86.7% mortality. *An. culicifacies* was susceptible to cyfluthrin in 5 districts and showed possible resistance (mortality – 92.4%–96.9%) in 5 districts (Figure 6B). The mortality correlation of DDT and cyfluthrin susceptibility among plain, rocky and hilly-forested topographies was significant at p < 0.05. For malathion and deltamethrin, the differences between the mortalities among the three topographies was not significant (see Supplementary Table 2).

### 3.9 The Northeastern states

**Figure 7:**
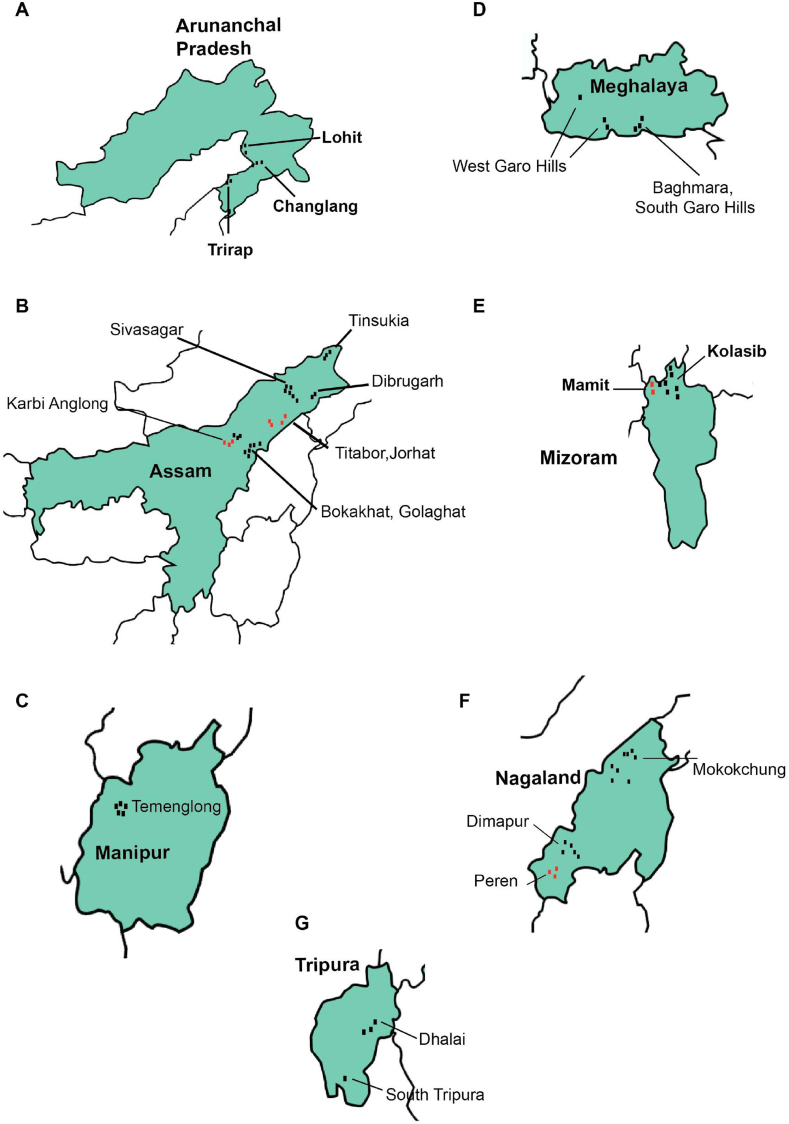
Maps of the Northeastern states showing the location of study districts in Arunachal Pradesh (A), Assam (B), Manipur (C), Meghalaya (D), Mizoram (E), Nagaland (F), and Tripura (G).

The study revealed that for DDT, *An. culicifacies* (n = 442) showed resistance in 2 study districts of Assam (Supplementary Table 1A), while *An. minimus* (n = 244) remained susceptible in 11 districts in 4 states (Supplementary Table 1C). The other vector *An. baimaii* (n = 365) was susceptible to DDT in 13 districts from 6 states (Supplementary Table 1C). The primary malaria vector species were completely susceptible except *An. culicifacies* in districts Udalgiri and Kamrup(R) of Assam with plain forest, foothill and plain ecotypes. *An. culicifacies* did not show any significant difference in mortality for DDT and malathion across the topographies at p < 0.05. The secondary malaria vector *An. annularis* (n = 2,310) was tested for insecticide susceptibility in 19 districts in 7 states. *An. annularis* was susceptible to DDT and deltamethrin, and it was susceptible to malathion in one district of Meghalaya and 2 districts of Tripura (Supplementary Table 1D). *An. philippinensis/nivepes* (n = 6,929) showed susceptibility to DDT, malathion and deltamethrin in 23 districts of the 5 states (Arunachal Pradesh, Assam, Meghalaya, Mizoram, and Nagaland) (Figure 7). *An. philippinensis/nivepes* was susceptible to fenitrothion, lambda-cyhalothrin, and permethrin in 13 districts of 4 states (Arunachal Pradesh, Assam, Mizoram, and Nagaland, Supplementary Table 1D). *An. aconitus* (n = 80) collected from 1 district each of Assam, Mizoram and Nagaland states was susceptible to DDT (Supplementary Table 1D).

To summarize, in the present study from 15 states of India, *An. culicifacies* was found to be resistant to DDT alone in 19 districts of 5 states. It was double resistant to DDT-malathion in 16 districts of 5 states, to malathion-deltamethrin in 2 districts of 1 state, and DDT-deltamethrin in 6 districts of 2 states. *An. culicifacies* was triple resistant to DDT-malathion-deltamethrin in 9 districts in 4 states. Four out of the fifteen states included in this study, i.e., Haryana, Jharkhand, Karnataka, and Maharashtra reported triple resistance. In contrast, *An. minimus* and *An. baimaii* that are vectors of prominence in Northeastern states [11] were susceptible to all the insecticides including DDT. *An. culicifacies* in Northeastern region was resistant to DDT in Assam.

## 4 Discussion

Genetic, biological, and operational factors influence the evolution of insecticide resistance in disease vectors [12, 13, 15]. The operational factors include coverage of interventions, the optimum dosage of application, and resultant selection pressure [14, 15]. Operational factors associated with interventions such as indoor residual spray with improper dosage and non-uniformity may lead to uneven coverage resulting in differential selection pressure. This might alter susceptibility levels in the same species in varied topographies, as observed in this study. Here we report the insecticide susceptibility status of the critical Indian malaria vectors in 328 villages within 79 districts from 15 states with varied ecotypes. In this study, *An. culicifacies* was found to be resistant to at least one insecticide in 52 districts (Supplementary Table 3). It showed variation in susceptibility categories (susceptible, possible, and confirmed resistance), indicating the dynamic status of insecticide resistance in the population. Further, significant differences were found between the ecotype and insecticide susceptibility to some insecticides in Jharkhand and Uttar Pradesh. Madhya Pradesh and other states did not show significant differences across the species in susceptibility. Topographies possibly influence the preponderance of a given species and behavioural differences. Previous studies on *An. culicifacies* sibling species (provisionally designated as A, B, C, D, and E) have suggested variations in sympatric, seasonal prevalence, host feeding preference, vector potential, and insecticide susceptibility [16, 17, 18, 19, 20]. Differential prevalence of sibling species in a given topography could be one of the reasons for the observed variations in susceptibility to insecticides.

In India, changes in the vector control policy have been brought over the years based on insecticide resistance data. DDT was introduced in IRS in the early 1950s, HCH in 1958, malathion in 1969, and synthetic pyrethroids in the mid 1990s [21]. Currently for adult vector control, insecticide DDT (organo chlorine), malathion (organophosphate) and deltamethrin, alpha-cypermethrin, and lambda-cyhalothrin (pyrethroids) are mostly used for indoor residual spray (IRS), and deltamethrin and alpha-cypermethrin for impregnated LLINs. For larval control temephos, bacterial pesticides (*Bacillus thuringiensis var. israelensis*, *B. sphaericus*), and IGR compounds (diflubenzuron, pyriproxyfen); and for space spray malathion (technical) and formulations of deltamethrin (pyrethroid)/natural pyrethrum extracts are in use. Vector control in India per se is control of *An. culicifacies* because of its wide distribution. It transmits nearly two-thirds of new malaria cases each year, and has also developed multiple resistance to different classes of insecticides [8]. The current vector control strategies rely on pyrethroids both for IRS and LLINs. Thus, there is an impending threat of the development of widespread pyrethroid resistance [8]. It was observed earlier in India that the critical reason for malaria resurgence in the mid-1970s was insecticide resistance in *An. culicifacies* [22]. This prompts for implementing vector control strategies with effective tools to evade or defer the onset of resistance [23]. In India, multiple insecticide resistance in vector species is observed, which may persist for a variable duration of time [12, 13, 14, 21]. Generally, an insecticide-unselected population will have insects that are fully susceptible with resistance gene in rarity, possible due to “fitness cost” [24].

The effective strategy suggested for managing insecticide resistance is rotation, mosaics, mixtures, and combinations of insecticides [25]. Though not in use in India at present, improved LLINs with synergist piperonyl butoxide and insecticide deltamethrin mixture on the roof, and deltamethrin in different concentrations on panels, and a mosaic of the same was reportedly effective for resistance management compared to deltamethrin alone net [26]. Another possibility for resistance management is using insecticide mixtures from different classes with different modes of action to promote the efficacy of either of the components [27]. Such products for IRS and LLINs, such as deltamethrin + clothianidin mixtures for IRS [28, 29, 30, 31, 32] and deltamethrin/permethrin + synergists for LLINs [33, 34, 35, 36] are getting available in India, though they are not registered as yet. The above interventions are of special importance in the run-up for malaria elimination as the vectors have developed multiple resistance to insecticides, including pyrethroids. The combination of two vector control tools is another management strategy used with the presumption that the mosquito should have the propensity for simultaneous exposure to both the vector control tools resulting in choice for the efficacy such as pyrethroid LLINs, and non-pyrethroid IRS.

In the present study *An. culicifacies* showed single resistance to DDT in 19 districts, double resistance to DDT-malathion in 16 districts, malathion-deltamethrin in 2 districts, to DDT-deltamethrin in 6 districts and triple resistance to DDT-malathion-deltamethrin in 9 districts. This presents an alarming scenario for India for malaria control. For example, the triple resistance to DDT, malathion, and deltamethrin in Haryana, Jharkhand, Karnataka, and Maharashtra needs urgent attention for resistance management. In contrast, *An. minimus* and *An. baimaii* (from Northeastern states) were susceptible to all the insecticides. However, a limitation of the present study in the Northeastern states was that we could not test *An*. *minimus* and *An*. *baimai* mosquitoes in sufficient numbers due to low density in the field in some sites, and therefore we could expose only a few mosquitoes. Other researchers have encountered this situation as well [6].

The Northeastern states had regular IRS with DDT since the initiation of the control programme in the 1950s and for about 2 decades in a combination with pyrethroid LLINs. The combination was very effective for the control of *An. minimus*. *An. baimaii* is an exophilic and exophagic mosquito by behaviour [37] and thus was devoid of continued insecticide selection pressure and possibly remained susceptible to all insecticides. Recently, *An. baimaii* has shown endophilic behavior but its epidemiological significance is yet to be ascertained [38]. Thus, there is a need for a renewed focus on malaria control in the Northeastern states. As India hurtles towards malaria elimination by 2030, it is vital that the gains so far are retained for the foreseeable future in the context of vector control. In areas with widespread multiple insecticide-resistant vectors, the mitigation option is resistance management by using the insecticides with alternative modes of action, different modes of deployment and to introduce new vector control tools when available. The strategy management should limit the geographical spread of resistance to new areas by using effective vector control tools focusing on novel insecticides and should target to mitigate the impact of resistance. The enzyme families being investigated for malaria parasites can be explored as potent drug targets for malaria vectors [39, 40, 41, 42]. It is important that data generated on insecticide resistance in vectors and data on other entomological surveillance indicators are regularly shared with the national malaria programme preferably via a digital platform [43]. The work presented here will help formulate policies that are urgently required to prevent the widespread dissemination of insecticide-resistant vectors.

## Declarations

### Author contribution statement

Kamaraju Raghavendra: Conceived and designed the experiments.

Manju Rahi: Designed the study, analyzed and interpreted the data and wrote the paper.

Vaishali Verma, Poonam Sharma Velamuri: Analyzed and interpreted the data; Wrote the paper.

Divya Kamaraju: Analyzed and interpreted the data.

Jyoti Chhibber-Goel: Analyzed and interpreted the data; Wrote the paper.

Kalpana Baruah: Provided national programme data, analysis tools or data.

Amit Sharma: Analyzed and interpreted the data, designed the flow of figures and wrote the paper.

### Funding statement

Dr Amit Sharma was supported by 10.13039/501100006143Department of Science and Technology [JCB41].

Jyoti Chhibber-Goel was supported by Department of Biotechnology [BT/PR30603/BIC/101/1104/2018].

### Data availability statement

Data included in article/supp. material/referenced in article.

### Declaration of interest's statement

The authors declare no conflict of interest.

### Additional information

No additional information is available for this paper.

## Appendix A Supplementary data

MMC1 : ir_tables_supp_180522_revision.docx MMC1 : ir_tables_supp_180522_revision.docx
