# Supplementary material for: Insecticide resistance status of malaria vectors in the malaria endemic states of India: implications and way forward for malaria elimination
Source: Heliyon. 2022 Nov 26;8(12):e11902. doi: 10.1016/j.heliyon.2022.e11902 (PMC9732330; doi:10.1016/j.heliyon.2022.e11902)
Supplement: MMC1 : ir_tables_supp_180522_revision.docx [file mmc1.docx]

**Supplementary Table 1a: Percent mortality and status of resistance to different insecticides in *An. culicifacies* in the selected districts of 9 states in India**

| State | District | Year | Insecticide % / Percentage Mortality (n) / Status | | | | | | | | | | | | | |
| --- | --- | --- | --- | --- | --- | --- | --- | --- | --- | --- | --- | --- | --- | --- | --- | --- |
|  |  |  | DDT 4% | Status | Malathion 5% | Status | Deltamethrin 0.05% | Status | Alphacypermethrin 0.05% | Status | Permethrin 0.75% | Status | Cyfluthrin0.15% | Status | Lambda cyhalothrin 0.05% | Status |
| Assam | Udalgiri | 2018 | 65.7 (105) | CR | 91.8 (102) | PR | 99.1 (115) | S | - | - | - | - | - | - | - | - |
|  | Kamrup (R) | 2018 | 67.5(80) | CR | 80(40) | CR | - | - | - | - | - | - | - | - | - | - |
| Gujarat | Panchmahal | 2019 | - | - | 75 (160) | CR | 85 (160) | CR | 95 (160) | PR | 90 (160) | PR | - | - | - | - |
|  | Kheda |  | - | - | 70 (160) | CR | 75 (160) | CR | 95 (160) | PR | 85 (160) | CR | - | - | - | - |
| Haryana | Nuh | 2019 | 38 (120) | CR | 78 (129) | CR | 86 (155) | CR | - | - | - | - | - | - | - | - |
|  | Palwal |  | 29 (165) | CR | 90 (86) | CR | 83 (150) | CR | - | - | - | - | - | - | - | - |
| Jharkhand | Giridih | 2019 | 17.5 (200) | CR | 89 (200) | CR | 86.5 (200) | CR | - | - | 93.5 (200) | PR | 93 (100) | PR | 85 (40) | CR |
|  | Palamu |  | 11.5 (200) | CR | 83.5 (200) | CR | 87.5 (200) | CR | - | - | 89 (200) | CR | 86.5 (200) | CR | 92 (200) | PR |
|  | Latehar |  | 15.5 (200) | CR | 88.5 (200) | CR | 93.5 (200) | PR | - | - | 93.5 (200) | PR | 92 (200) | PR | 86 (200) | CR |
|  | Koderma |  | 9.5 (200) | CR | 93 (200) | PR | 85 (200) | CR | - | - | 89 (200) | CR | 51 (200) | CR | 79.5 (200) | CR |
|  | Chatra |  | 4.5 (200) | CR | 87 (200) | CR | 58.5 (200) | CR | - | - | 65.5 (200) | CR | 40 (100) | CR | 57 (100) | CR |
|  | Dhanbad |  | 12 (200) | CR | 89.5 (200) | CR | 70.5 (200) | CR | - | - | 94 (100) | PR | - | - | - | - |
|  | Gumla | 2018 | 8.3 (157) | CR | 100 (165) | S | 100 (160) | S | - | - | 100 (162) | S | - | - | - | - |
|  | Khunti |  | 7.5 (180) | CR | 100 (160) | S | 85 (200) | CR | - | - | 100 (155) | S | - | - | - | - |
|  | West Singhbhum |  | 47 (160) | CR | 69 (200) | CR | 84 (200) | CR | - | - | 85.6 (160) | CR | - | - | - | - |
|  | Godda |  | 10 (200) | CR | - | - | 76 (180) | CR | - | - | 93.4 (180) | PR | - | - | - | - |
|  | Sahibganj |  | 23.5 (200) | CR | 98 (100) | S | 77.7 (145) | CR | - | - | 95.5 (175) | PR | - | - | - | - |
|  | Simdega | 2017 | 25.3 (180) | CR | 99 (180) | S | 85 (200) | CR | - | - | 79.3 (155) | CR | - | - | - | - |
| Karnataka | Kalaburgi | 2019 | 2 (50) | CR | 49 (51) | CR | 86.5 (52) | CR | 16 (50) | CR | - | - | - | - | - | - |
| Madhya Pradesh | Sidhi | 2019 | 27.6 (105) | CR | 65.3 (150) | CR | 98.3 (180) | S | 96.7 (180) | PR | - | - | - | - | - | - |
|  | Datiya |  | 60 (100) | CR | 93 (100) | PR | 100 (100) | S | 97 (100) | PR | - | - | - | - | - | - |
|  | Shivpuri |  | 41 (100) | CR | 87 (100) | CR | 97 (100) | PR | 93 (105) | PR | - | - | - | - | - | - |
|  | Bhind |  | 16.2 (105) | CR | 63.8 (105) | CR | 97.1 (105) | PR | 95.2 (105) | PR | - | - | - | - | - | - |
|  | Hoshangabad | 2018 | 7.6 (105) | CR | 72.4 (105) | CR | 95.2 (105) | PR | 96.2 (105) | PR | - | - | - | - | - | - |
|  | Khargone |  | 12.5 (120) | CR | 65 (120) | CR | 96.7 (120) | PR | 93.3 (120) | PR | - | - | - | - | - | - |
|  | Alirajpur |  | 7.6 (105) | CR | 58.1 (105) | CR | 96.2 (105) | PR | 87.6 (105) | CR | - | - | - | - | - | - |
|  | Dhar |  | 17.5 (120) | CR | 65 (120) | CR | 93.3 (120) | PR | 84.2 (120) | CR | - | - | - | - | - | - |
|  | Singrouli | 2017 | 18.1 (105) | CR | 70.5 (105) | CR | 93.3 (105) | PR | 90.5 (105) | PR | - | - | - | - | - | - |
|  | Umaria |  | 14.3 (105) | CR | 76.2 (105) | CR | 96.2 (105) | PR | 95.2 (105) | PR | - | - | - | - | - | - |
|  | Anuppur |  | 16.2 (105) | CR | 64.8 (105) | CR | 98.1 (105) | S | 98.1(105) | S | - | - | - | - | - | - |
|  | Panna |  | 12.4 (105) | CR | 73.3 (105) | CR | 99 (105) | S | 99 (105) | S | - | - | - | - | - | - |
|  | Tikamgarh |  | 16.2 (105) | CR | 84.8 (105) | CR | 100 (105) | S | 100 (105) | S | - | - | - | - | - | - |
| Maharashtra | Gadchiroli | 2019 | 37 (227) | CR | 71.9 (196) | CR | 87.8 (255) | CR | 88.2 (110) | CR | 92.2 (90) | PR | 81 (274) | CR | 70.1 (338) | CR |
| Odisha | Kalahandi | 2020 | 33 (120) | CR | 71 (120) | CR | 92.5 (120) | PR | - | - | - | - | 73 (120) | CR | - | - |
|  | Angul |  | 28 (120) | CR | 98 (120) | S | 95.2 (120) | PR | - | - | - | - | 91.4 (120) | PR | - | - |
|  | Cuttack | 2017-18 | 60 (30) | CR | - | - | - | - | - |  |  |  | 83.3 (30) | CR |  |  |
|  | Khurda |  | 56.6 (30) | CR | - | - | - | - | - |  |  |  | 66.6 (30) | CR |  |  |
|  | Puri |  | 60 (30) | CR | - | - | - | - | - |  |  |  | 70 (30) | CR |  |  |
|  | Bhadrak |  | 50 (30) | CR | - | - | - | - | - |  |  |  | 63.3 (30) | CR |  |  |
|  | Jagatsinghpur |  | 46.6 (30) | CR | - | - | - | - | - |  |  |  | 53.3 (30) | CR |  |  |
|  | Jajpur |  | 60 (30) | CR | - | - | - | - | - |  |  |  | 73.3 (30) | CR |  |  |
|  | Mayurbhanj |  | 40 (30) | CR | - | - | - | - | - |  |  |  | 50 (30) | CR |  |  |
| Uttar Pradesh | Prayagraj | 2019 | 0 (160) | CR | 93.3 (160) | PR | 91.9 (160) | PR | - | - | - | - | 95.6 (160) | PR | - | - |
|  | Mirzapur |  | 4.4 (160) | CR | 94.4 (160) | PR | 96.3 (160) | PR | - | - | - | - | 96.9 (160) | PR | - | - |
|  | Sonebhadra |  | 3.9 (180) | CR | 92.2 (180) | PR | 91.7 (180) | PR | - | - | - | - | 92.2 (180) | PR | - | - |
|  | Hathras | 2018 | 0 (120) | CR | 95 (120) | PR | 96.7 (120) | PR | - | - | - | - | 100 (120) | S | - | - |
|  | Gautam Budh Nagar |  | 3.3 (60) | CR | 80 (60) | CR | 91.7 (60) | PR | - | - | - | - | 100 (60) | S | - | - |
|  | Jhansi |  | 2.5 (120) | CR | 94.2 (120) | PR | 92.5 (120) | PR | - | - | - | - | 98.3 (120) | S | - | - |
|  | Banda |  | 4.2 (120) | CR | 95.8 (120) | PR | 86.7 (120) | CR | - | - | - | - | 96.7 (120) | PR | - | - |
|  | Kanpur Dehat |  | 5.8 (120) | CR | 96.7 (120) | PR | 93.3 (120) | PR | - | - | - | - | 96.7 (120) | PR | - | - |
|  | Saharanpur | 2017 | 6.9 (130) | CR | 93.1 (130) | PR | 91.5 (130) | PR | - | - | - | - | 100 (120) | S | - | - |
|  | Badaun |  | 0 (90) | CR | 98.9 (90) | S | 92.2 (90) | PR | - | - | - | - | 100 (90) | S | - | - |
|  |  |  |  |  |  |  |  |  |  |  |  |  |  |  |  |  |

(n) – number exposed ; S- Susceptible (98-100% Mortality); PR- Possible Resistance (90-97% Mortality); CR- Confirmed Resistance (< 90% Mortality).

**Supplementary Table 1b: Percent mortality and status of resistance to different insecticides in *An. stephensi* (1 state) and *An. fluviatilis* (2 states) in the select districts of India**

| S. No. | Species | | State | | District | | | Year | | Insecticide % / Percentage Mortality (n) / Status | | | | | | | | | | | | | | | | | | | |  |  |
| --- | --- | --- | --- | --- | --- | --- | --- | --- | --- | --- | --- | --- | --- | --- | --- | --- | --- | --- | --- | --- | --- | --- | --- | --- | --- | --- | --- | --- | --- | --- | --- |
|  |  |  |  |  |  |  |  |  |  | DDT 4% | | | Status | Malathion 5% | | Status | | Deltamethrin 0.05% | | Status | | Permethrin 0.75% | | Status | | Cyfluthrin0.15% | | Status | |  |  |
| 1 | *An. stephensi*(Primary vector) | | Haryana | | Nuh | | | 2019 | | 31 (100) | | | CR | 69 (80) | | CR | | 85 (166) | | CR | | - | | - | | - | | - | |  |  |
|  |  |  |  |  | Palwal | | |  |  | 48 (110) | | | CR | 81 (120) | | CR | | 72 (219) | | CR | | - | | - | | - | | - | |  |  |
| 2 | *An. fluviatilis*(Primary vector) | | Jharkhand | | Giridih | | | 2019 | | 70 (40) | | | CR | ^-^ | | - | | - | | - | | - | | - | | - | | - | |  |  |
|  |  |  |  |  | Palamu | | |  |  | 75 (40) | | | CR | ^-^ | | - | | - | | - | | - | | - | | - | | - | |  |  |
|  |  |  |  |  | Latehar | | |  |  | 80 (20) | | | CR | ^-^ | | - | | - | | - | | - | | - | | - | | - | |  |  |
|  |  |  |  |  | Simdega | | | 2018 | | 29.5 (160) | | | CR | 100 (140) | | S | | 100 (120) | | S | | 100 (110) | | S | | - | | - | |  |  |
|  |  |  |  |  | Gumla | | |  |  | 40.2 (130) | | | CR | 98.4 (120) | | S | | 100 (120) | | S | | 100 (120) | | S | | - | | - | |  |  |
|  |  |  |  |  | Khunti | | |  |  | 45 (120) | | | CR | 100 (100) | | S | | 100 (115) | | S | | 100 (105) | | S | | - | | - | |  |  |
|  |  |  |  |  | West Singhbhum | | |  |  | 55.9 (175) | | | CR | 100 (155) | | S | | 99.3 (145) | | S | | 100 (140) | | S | | - | | - | |  |  |
|  |  |  |  |  | Godda | | |  |  | 38.2 (130) | | | CR | 100 (135) | | S | | 100 (140) | | S | | 100 (125) | | S | | - | | - | |  |  |
|  |  |  |  |  | Sahibganj | | |  |  | 18.5 (115) | | | CR | 100 (110) | | S | | 100 (110) | | S | | 100 (115) | | S | | - | | - | |  |  |
|  |  |  | Odisha | | Kalahandi | | | 2019 | | 90 (100) | | | PR | 98.5 (100) | | S | | 99 (100) | | S | | - | | - | | 98 (100) | | S | |  |  |
|  |  |  |  |  | Angul | | |  |  | 91 (100) | | | PR | 100 (100) | | S | | 100 (100) | | S | | - | | - | | 100 (100) | | S | |  |  |
|  | |  | |  | |  |  | |  | |  |  | | |  | |  | |  | |  | |  | |  | |  | |  | |  |

(n) – number exposed ; S- Susceptible (98-100% Mortality); PR- Possible Resistance (90-97% Mortality); CR- Confirmed Resistance (< 90% Mortality).

**Supplementary Table 1c: Percent mortality and status of resistance to different insecticides in *An. minimus* (5 states) and *An. baimaii* (6 states) in the select districts of India**

| S. No. | | Species | | State | | District | | | Year | | Percentage Mortality (n) | | | | | | | | | | | | | | | | |  |  |  |
| --- | --- | --- | --- | --- | --- | --- | --- | --- | --- | --- | --- | --- | --- | --- | --- | --- | --- | --- | --- | --- | --- | --- | --- | --- | --- | --- | --- | --- | --- | --- |
|  |  |  |  |  |  |  |  |  |  |  | DDT 4% | | Status | Malathion 5% | | | Status | Deltamethrin 0.05% | | | Status | | Permethrin 0.75% | | | Status | |  |  |  |
| 1 | | *An. minimus*(Primary vector) | | Arunachal Pradesh | | Changlang | | | 2019 | | 100 (40) | | S | - | | | - | - | | | - | | - | | | - | |  |  |  |
|  |  |  |  | Assam | | Dibrugarh | | | 2019 | | 100 (60) | | S | - | | | - | - | | | - | | - | | | - | |  |  |  |
|  |  |  |  |  |  | Golaghat | | |  |  | 100 (20) | | S | - | | | - | - | | | - | | - | | | - | |  |  |  |
|  |  |  |  |  |  | KarbiAnglong | | |  |  | 100 (37) | | S | - | | | - | - | | | - | | - | | | - | |  |  |  |
|  |  |  |  |  |  | Udalguri | | |  |  | 100 (10) | | S | - | | | - | - | | | - | | - | | | - | |  |  |  |
|  |  |  |  |  |  | Kokrazhar | | | 2018 | | 100 (15) | | S | - | | | - | - | | | - | | - | | | - | |  |  |  |
|  |  |  |  |  |  | Kamrup (M) | | | 2017 | | 100 (12) | | S | - | | | - | - | | | - | | - | | | - | |  |  |  |
|  |  |  |  | Jharkhand | | West Singhbhum | | | 2018 | | 96.7 (30) | | PR | 100 (30) | | | S | 100 (30) | | | S | | 100 (30) | | | S | |  |  |  |
|  |  |  |  | Meghalaya | | South Garo hills | | | 2019 | | 100 (10) | | S | - | | | - | - | | | - | | - | | | - | |  |  |  |
|  |  |  |  |  |  | East Garo hills | | | 2017 | | 100 (10) | | S | - | | | - | - | | | - | | - | | | - | |  |  |  |
|  |  |  |  | Mizoram | | Kolasib | | | 2019 | | 100 (10) | | S | - | | | - | - | | | - | | - | | | - | |  |  |  |
|  |  |  |  |  |  | Mamit | | |  |  | 100 (20) | | S | - | | | - | - | | | - | | - | | | - | |  |  |  |
| 2 | | *An. baimaii*(Primary vector) | | Assam | | Dibrugarh | | | 2019 | | 100 (40) | | S | - | | | - | - | | | - | | - | | | - | |  |  |  |
|  |  |  |  |  |  | Golaghat | | |  |  | 100 (20) | | S | - | | | - | - | | | - | | - | | | - | |  |  |  |
|  |  |  |  |  |  | KarbiAnglong | | |  |  | 100 (40) | | S | - | | | - | - | | | - | | - | | | - | |  |  |  |
|  |  |  |  |  |  | Udalguri | | | 2018 | | 100 (10) | | S | - | | | - | - | | | - | | - | | | - | |  |  |  |
|  |  |  |  | Arunachal Pradesh | | Changlang | | | 2019 | | 100 (40) | | S | - | | | - | - | | | - | | - | | | - | |  |  |  |
|  |  |  |  |  |  | Namsai | | |  |  | 100 (30) | | S | - | | | - | - | | | - | | - | | | - | |  |  |  |
|  |  |  |  | Nagaland | | Mokokchung | | | 2019 | | 100 (20) | | S | - | | | - | - | | | - | | - | | | - | |  |  |  |
|  |  |  |  | Meghalaya | | South Garo Hill | | | 2019 | | 100 (10) | | S | 100 (10) | | | S |  | | | - | | - | | | - | |  |  |  |
|  |  |  |  |  |  | East Garo Hill | | | 2017 | | 100 (20) | | S | - | | | - | - | | | - | | - | | | - | |  |  |  |
|  |  |  |  | Mizoram | | Kolasib | | | 2019 | | 100 (30) | | S | - | | | - | - | | | - | | - | | | - | |  |  |  |
|  |  |  |  |  |  | Mamit | | |  |  | 100 (50) | | S | - | | | - | - | | | - | | - | | | - | |  |  |  |
|  |  |  |  | Tripura | | South Tripura | | | 2019 | | 100 (15) | | S | 100 (10) | | | S |  | | | - | | - | | | - | |  |  |  |
|  |  |  |  |  |  | Dhalai | | | 2019 | | 100 (20) | | S | - | | | - | - | | | - | | - | | | - | |  |  |  |
|  | |  | |  | |  | |  |  | |  | |  | | |  |  | | |  |  | |  | |  |  | |  | |  |

S- Susceptible (98-100% Mortality); PR- Possible Resistance (90-97% Mortality); CR- Confirmed Resistance (< 90% Mortality)

**Supplementary Table 1d: Percent mortality and status of resistance to different insecticides in *An. annularis* (8 states), *An. philippinensi*s/nivipes (5 states) and *An. aconitus* (3 states) in the select districts of India**

| S. No. | Species | State | District | Year | Percentage Mortality (n) | | | | | | | | | | | |
| --- | --- | --- | --- | --- | --- | --- | --- | --- | --- | --- | --- | --- | --- | --- | --- | --- |
|  |  |  |  |  | DDT 4% | Status | Malathion 5% | Status | Deltamethrin 0.05% | Status | Permethrin 0.75% | Status | Lambda cyhalothrin 0.05% | Status | Fenitrothion 1% | Status |
| 1 | *An. annularis*(Secondary vector) | Arunachal Pradesh | Changlang | 2019 | 100 (60) | S | - | - | 100 (60) | S | - | - | - | - | - | - |
|  |  |  | Namsai |  | 100 (60) | S | - | - | 100 (60) | S | - | - | - | - | - | - |
|  |  |  | Tirap |  | 100 (60) | S | - | - | 100 (60) | S | - | - | - | - | - | - |
|  |  | Assam | Dibrugarh | 2019 | 100 (60) | S | - | - | 100 (60) | S | - | - | - | - | - | - |
|  |  |  | Tinsukia |  | 100 (60) | S | - | - | 100(60) | S | - | - | - | - | - | - |
|  |  |  | Golaghat |  | 100 (60) | S | - | - | 100 (60) | S | - | - | - | - | - | - |
|  |  |  | Jorhat |  | 100 (60) | S | - | - | 100 (60) | S | - | - | - | - | - | - |
|  |  |  | KarbiAnglong |  | 98.3 (60) | S | - | - | 100 (60) | S | - | - | - | - | - | - |
|  |  |  | Sivasagar |  | 100 (60) | S | - | - | 100 (60) | S | - | - | - | - | - | - |
|  |  | Jharkhand | Gumla | 2018 | 1.7 (110) | CR | 97.5 (110) | PR | 100 (95) | S | 100 (95) | S | - | - | - | - |
|  |  |  | Khunti |  | 0 (110) | CR | 97.5 (105) | PR | 99 (100) | S | 100 (100) | S | - | - | - | - |
|  |  |  | West Singhbhum |  | 2.9 (120) | CR | 100 (110) | S | 99.2 (110) | S | 100 (105) | S | - | - | - | - |
|  |  |  | Godda |  | 1.7 (110) | CR | 100 (95) | S | 100 (100) | S | 100 (100) | S | - | - | - | - |
|  |  |  | Sahibganj |  | 0 (115) | CR | 96.7 (105) | PR | 100 (110) | S | 100 (110) | S | - | - | - | - |
|  |  |  | Simdega | 2017 | 3 (100) | CR | 99 (100) | S | 100 (95) | S | 100 (90) | S | - | - | - | - |
|  |  | Manipur | Temenglong | 2019 | 100 (60) | S | - | - | 100 (60) | S | - | - | - | - | - | - |
|  |  | Meghalaya | West Garo Hills | 2019 | 100 (60) | S | - | - | 100 (60) | S | - | - | - | - | - | - |
|  |  |  | South Garo Hills |  | 100 (60) | S | 100 (10) | S | 100 (60) | S | - | - | - | - | - | - |
|  |  | Mizoram | Kolasib | 2019 | 100 (60) | S | - | - | 100 (60) | S | - | - | - | - | - | - |
|  |  |  | Mamit |  | 100 (60) | S | - | - | 100 (60) | S | - | - | - | - | - | - |
|  |  | Nagaland | Mokokchung | 2019 | 100 (60) | S | - | - | 100 (60) | S | - | - | - | - | - | - |
|  |  |  | Dimapur |  | 100 (60) | S | - | - | 100 (60) | S | - | - | - | - | - | - |
|  |  |  | Peren |  | 100 (60) | S | - | - | 100 (60) | S | - | - | - | - | - | - |
|  |  | Tripura | Dhalai | 2019 | 100 (60) | S | 100(10) | S | 100 (60) | S | - | - | - | - | - | - |
|  |  |  | South Tripura |  | 100 (60) | S | 100(10) | S | 100 (60) | S | - | - | - | - | - | - |
| 2 | *An. philippinensis/nivipes*(Secondary vector) | Arunachal Pradesh | Changlang | 2019 | 100 (60) | S | 100 (60) | S | 100 (60) | S | 100 (60) | S | 100 (60) | S | 100 (60) | S |
|  |  |  | Namsai |  | 100 (60) | S | 100 (60) | S | 100 (60) | S | 100 (60) | S | 100 (60) | S | 100 (60) | S |
|  |  |  | Tirap |  | 98.3 (60) | S | 100 (60) | S | 100 (60) | S | 100 (60) | S | 100 (60) | S | 100 (60) | S |
|  |  | Assam | Dibrugarh | 2019 | 100 (60) | S | 100 (60) | S | 98.3 (60) | S | 100 (60) | S | 100 (60) | S | 100 (60) | S |
|  |  |  | Tinsukia |  | 100 (60) | S | 100 (60) | S | 98.3 (60) | S | 100 (60) | S | 100 (60) | S | 100 (60) | S |
|  |  |  | Golaghat |  | 100 (60) | S | 100 (60) | S | 100 (60) | S | 100 (60) | S | 100 (60) | S | 100 (60) | S |
|  |  |  | KarbiAnglong |  | 100 (60) | S | 100 (60) | S | 98.3 (60) | S | 100 (60) | S | 100 (60) | S | 100 (60) | S |
|  |  |  | Sivasagar |  | 100 (60) | S | 98.3 (60) | S | 100 (60) | S | 100 (60) | S | 100 (60) | S | 100 (60) | S |
|  |  |  | Kokrazhar | 2018 | 98.8 (85) | S | 100 (80) | S | 100 (40) | S | - | - | - | - | - | - |
|  |  |  | Kamrup (M) |  | 98.1 (103) | S | 98 (100) | S | 100 (110) | S | - | - | - | - | - | - |
|  |  |  | Bongaigaon |  | 100 (20) | S | 100 (20) | S | 100 (10) | S | - | - | - | - | - | - |
|  |  |  | Goalpara |  | 100 (90) | S | 100 (74) | S | 98.8 (85) | S | - | - | - | - | - | - |
|  |  |  | Kamrup (R) |  | 100 (80) | S | 100 (40) | S | 98.1 (103) | S | - | - | - | - | - | - |
|  |  |  | Nalbari |  | 98 (100) | S | 100 (110) | S | 99.1 (110) | S | - | - | - | - | - | - |
|  |  |  | Baksa |  | 98 (100) | S | 100 (100) | S | 100 (20) | S | - | - | - | - | - | - |
|  |  |  | Udalguri | 2017 | 99.1 (110) | S | 98 (100) | S | 100 (100) | S | - | - | - | - | - | - |
|  |  | Nagaland | Mokokchung | 2019 | 100 (60) | S | 100 (60) | S | 100 (60) | S | 100 (60) | S | 100 (60) | S | 100 (60) | S |
|  |  |  | Dimapur |  | 100 (60) | S | 100 (60) | S | 100 (60) | S | 100 (60) | S | 100 (60) | S | 100 (60) | S |
|  |  |  | Peren |  | 100 (60) | S | 100 (60) | S | 100 (60) | S | 100 (60) | S | 100 (60) | S | 100 (60) | S |
|  |  | Meghalaya | Ri Bhoi | 2018 | 100 (20) | S | 100 (10) | S | 100 (90) | S | - | - | - | - | - | - |
|  |  |  | South West Garo Hill |  | 100 (74) | S | 98.8 (85) | S | 100 (80) | S | - | - | - | - | - | - |
|  |  | Mizoram | Kolasib | 2019 | 98.3 (60) | S | 100 (60) | S | 100 (60) | S | 100 (60) | S | 100 (60) | S | 100 (60) | S |
|  |  |  | Mamit |  | 100 (60) | S | 100 (60) | S | 100 (60) | S | 100 (60) | S | 100 (60) | S | 100 (60) | S |
| 3 | *An. aconitus*(Secondary vector) | Assam | Golaghat | 2019 | 100 (20) | S | - | - | - | - | - | - | - | - | - | - |
|  |  | Nagaland | Mokokchung | 2019 | 100 (40) | S | - | - | - | - | - | - | - | - | - | - |
|  |  | Mizoram | Kolasib | 2019 | 100 (20) | S | - | - | - | - | - | - | - | - | - | - |

S- Susceptible (98-100% Mortality); PR- Possible Resistance (90-97% Mortality); CR- Confirmed Resistance (< 90% Mortality)

**Supplementary Table 2: Association of mortalities in *An. culicifacies* to different insecticides and topography in India**

| State | District | Fisher exact test, p value (p < .05) | |
| --- | --- | --- | --- |
|  |  | Significant | Not Significant |
| **Jharkhand** | **Plain and Hilltop** | | |
|  | Simdega | Permethrin - 0.75%,p < 0.00001* | DDT, MLN and DLM |
|  | Gumla | DDT - 4%, p =0.0073*, | MLN, DLM and PM |
|  | Khunti | DDT - 4%, p =0.0069*,  Deltamethrin - 0.05%, p = 0.0281* | MLN and PM |
|  | **Plain and Forested** | | |
|  | Palamu | Permethrin - 0.75%,p = 0.04* | DDT, MLN, DLM, CYF and LC |
|  | Koderma | Malathion - 5%, p = 0.0101* | DDT, DLM,PM, CYF and LC |
|  | **Plain and Foothill** | | |
|  | Sahibganj | DDT - 4%, p< 0.00001*,  Permethrin - 0.75%,p = 0.0432* | DLM |
|  | Giridih | Deltamethrin - 0.05%, 0.0119* | DDT, MLN and PM |
|  | Chatra | Malathion - 5%, p = 0.0193* | DDT, DLM and PM |
|  | Dhanbad | Deltamethrin - 0.05%, p = 0.0128* | DDT and MLN |
|  | **Forested and Hilly** | | |
|  | West Singhbhum | DDT - 4%, p = 0.0017*,  Malathion - 5%, p < 0.00001*,  Deltamethrin - 0.05%, p< 0.00001*,  Permethrin - 0.75%, p < 0.00001* |  |
|  | **Forested and Foothills** | | |
|  | Godda | Permethrin - 0.75%, p = 0.007* | DDT and DLM |
|  | Latehar | Deltamethrin - 0.05%, p = 0.0025* | DDT, MLN, PM, CYF and LC |
| **Haryana** | **Plain** |  |  |
|  | Nuh and Palwal | Malathion - 5%, p =.01711 | DDT and DLM |
| **Uttar Pradesh** | **Plains**(Gautam Budh Nagar, Saharanpur, Badaun, Hathras, Jhansi, Banda, Kanpur Dehat)**, Rocky** (Prayag Raj) **and Hilly Forested** (Mirzapur and Sonebhadra) | | |
|  |  | DDT - 4%, p =0.041*,  CYF-0.15%, p < 0.00001 | MLN and DLM |
|  | **Plains** (Gautam Budh Nagar, Saharanpur, Badaun, Hathras, Jhansi, Banda, Kanpur Dehat) **and Rocky** (Prayag Raj) | | |
|  |  | DDT - 4%, p = 0.017*,  CYF-0.15%, p=.038538 | MLN and DLM |
|  | **Plains** (Gautam Budh Nagar, Saharanpur, Badaun, Hathras, Jhansi, Banda, Kanpur Dehat) **and Hilly Forested** (Mirzapur and Sonebhadra) | | |
|  |  | CYF-0.15%, p < 0.00001 | DDT, MLN and DLM |
|  | **Rocky** (Prayag Raj) **and Hilly Forested** (Mirzapur and Sonebhadra) | | |
|  |  | DDT - 4%, p = 0.009*,  CYF-0.15%, p < 0.00001 | MLN and DLM |

*p < .05

MLN = malathion, DLM = deltamethrin, PM = permethrin, CYF = cyfluthrin and LC = λ-cyhalothrin

**Supplementary Table 3: Susceptibility status in *An. culicifacies* to 3 insecticides in 52 districts* in India**

| **Insecticides** | **Resistance status** | | | **ND** |
| --- | --- | --- | --- | --- |
|  | **S** | **PR** | **CR** |  |
| **DDT** | 0 | 0 | 50 | 2 |
| **Malathion** | 6 | 11 | 27 | 8 |
| **Deltamethrin** | 7 | 20 | 17 | 8 |

S- Susceptible (98-100% Mortality); PR- Possible Resistance (90-97% Mortality); CR- Confirmed Resistance (> 90% Mortality); ND- Not Done

* Districts in 9 states and 2 districts of Assam
